# The Promising Success of Project Extension for Community Healthcare Outcomes (ECHO) Diabetes: Case Series

**DOI:** 10.2196/46050

**Published:** 2023-08-03

**Authors:** Lauren Figg, Ananta Addala, Ishaan Jain, Claudia Anez, Paul Midney, Corin DeChirico, Colleen Symanski, Brian C Fitzgerald, Kristi Colbert, Terry Raymer, Candy Stockton-Joreteg, Elizabeth Murphy, Leah Collins, Cyd Bernstein, Melanie Hechavarria, Eleni P Sheehan, Angelina Bernier, Sarah C Westen, Korey K Hood, Dessi P Zaharieva, Marina Basina, Nicolas Cuttriss, Stephanie L Filipp, Matthew J Gurka, Ashby F Walker, David M Maahs, Michael J Haller, Rayhan A Lal

**Affiliations:** 1 Division of Endocrinology Department of Pediatrics Stanford University School of Medicine Stanford, CA United States; 2 Stanford Diabetes Research Center Stanford University School of Medicine Stanford, CA United States; 3 Stanford University Stanford, CA United States; 4 Division of Endocrinology Department of Pediatrics University of Florida College of Medicine Gainesville, FL United States; 5 University of Florida Diabetes Institute Gainesville, FL United States; 6 Healthcare Network of Southwest Florida Naples, FL United States; 7 Treasure Coast Community Health Center Vero Beach, FL United States; 8 University of Florida Health Family Medicine Old Town, FL United States; 9 United Indian Health Services Potawot Health Village Arcata, CA United States; 10 Humboldt Independent Practice Association Eureka, CA United States; 11 Valley Diabetes & Obesity Modesto, CA United States; 12 Anderson Valley Health Center Boonville, CA United States; 13 Department of Clinical and Health Psychology University of Florida Gainesville, FL United States; 14 Division of Endocrinology Department of Medicine Stanford University School of Medicine Stanford, CA United States; 15 Extension for Community Healthcare Outcomes Diabetes Action Network Chevy Chase, MD United States; 16 Department of Pediatrics University of Florida College of Medicine Gainesville, FL United States; 17 Department of Health Services Research, Management and Policy University of Florida Gainesville, FL United States

**Keywords:** type 1 diabetes, care delivery, primary care, community health care

## Abstract

**Background:**

In the United States, there are over 37 million people with diabetes but only 8000 endocrinologists. Therefore, many people with diabetes receive care exclusively from primary care providers (PCPs). To democratize knowledge regarding insulin-requiring diabetes through tele-education, Stanford University and the University of Florida developed Project Extension for Community Healthcare Outcomes (ECHO) Diabetes.

**Objective:**

ECHO Diabetes uses a Hub and Spoke model connecting specialists (the “Hub”) with PCPs (the “Spokes”). One-hour, weekly sessions include Hub diabetes didactic presentations and Spoke deidentified case presentations. Lessons learned during these sessions target provider knowledge and confidence surrounding diabetes management and patient care.

**Methods:**

Spokes were asked to provide short descriptions of people with diabetes whose diabetes management improved directly or indirectly from their providers’ participation or their involvement with a Diabetes Support Coach (DSC). We provide a case series to describe individuals and outcomes. Because this study was not a randomized controlled trial and was a prospective observation of patients with the intervention delivered to providers, the trial is not registered in a public trials registry.

**Results:**

A case series of 11 people with diabetes was compiled from 10 PCPs and 1 DSC from California and Florida between 2021 and 2022. The principal impact of ECHO Diabetes is the education amplified from PCPs and DSCs to people with diabetes. In all cases, people with diabetes reported increased engagement and improved diabetes management. Several cases reflected increased access to diabetes technology, improvement in glycemic outcomes, and positive trends in mental health measures.

**Conclusions:**

This case series elucidates the potential value of the ECHO Diabetes program to people with diabetes who receive their diabetes care from PCPs. Those matched with a DSC saw clinically significant improvements in hemoglobin A_1c_ and mental health outcomes.

## Introduction

**Figure 1:**
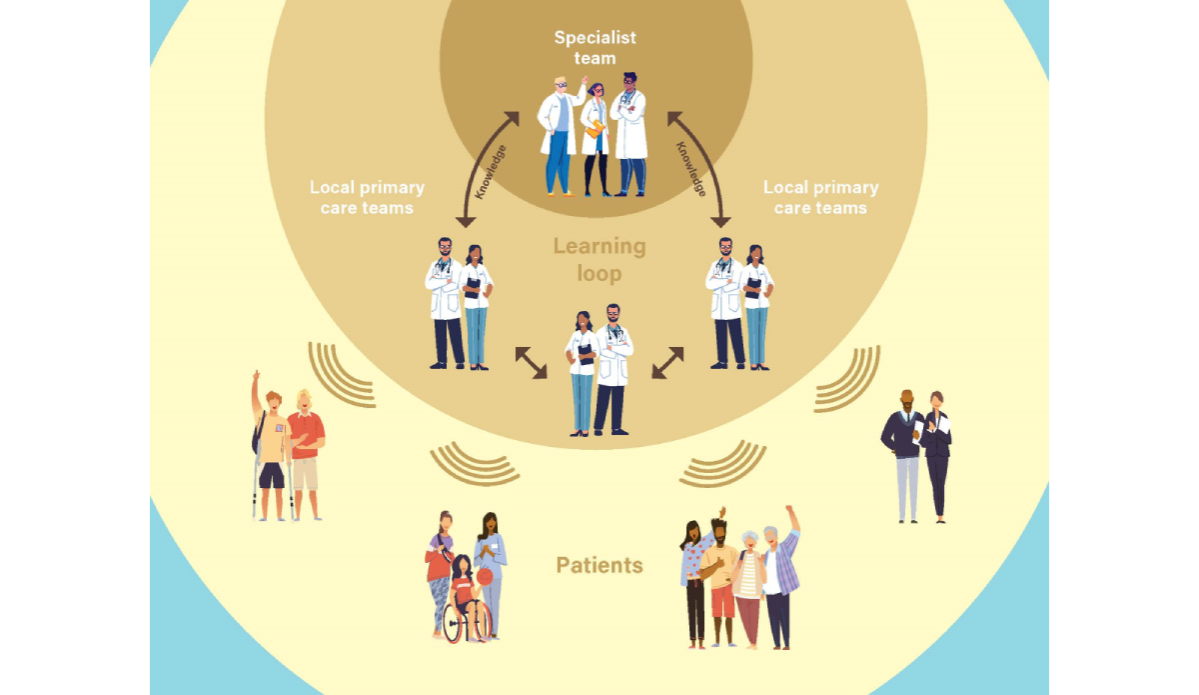
Project Extension for Community Healthcare Outcomes overview.

While more than 37 million Americans live with diabetes [1], there are only around 8000 endocrinologists, 1 for every 4625 people with diabetes, in the United States [2]. Furthermore, as endocrinologists are not distributed equitably and many do not provide care for people with diabetes [3], numerous people with diabetes receive care exclusively from primary care providers (PCPs). The Extension for Community Healthcare Outcomes (ECHO) model created at the University of New Mexico empowers PCPs through the use of tele-education. The model uses videoconferencing technology to connect community PCPs to subject matter experts at an academic medical center. Rather than patients remotely connecting to a specialist, the model facilitates education from specialists to PCPs in rural and underserved communities on topics related to chronic disease management in the form of regular, 1-hour didactic presentations and building a community of practice. Additionally, PCPs have opportunities to present case presentations on their own patients and receive feedback and support from both the subject matter experts and the peer providers who attend the sessions. PCPs develop subspecialty expertise over time and can become valuable resources in their communities for people with chronic medical conditions. The model aims to amplify and democratize specialty knowledge to improve health equity and outcomes in the communities it serves. Figure 1 highlights the ECHO model and its reach from academic medical centers, to community PCPs, to patients with complex chronic medical conditions. The Project ECHO model was designed to improve management practices and the overall quality of life (QOL) for people who lack access to specialists or routine care in underserved or rural communities and has been adapted in many disease types including hepatitis C, chronic pain, rheumatologic disorders, and behavioral health [4,5]. Stanford University and the University of Florida have partnered with Project ECHO to develop this model for democratizing specialty knowledge regarding insulin-requiring diabetes [6-11]. Through this program, the multidisciplinary Project ECHO Hub team offers real-time support and frequent presentations on topics related to diabetes treatment to PCPs.

Project ECHO Diabetes conducted outreach to primary care clinics and Federally Qualified Health Centers in high-need catchment areas in California and Florida, the locations of the respective universities. To identify these high-need catchment areas, the Neighborhood Deprivation Index was used along with geocoding of PCPs and endocrinologists in each state to identify areas with low access to endocrinology providers and high health risk or poverty [9]. Site visits, advertisements in family medicine publications, and informational web-based sessions were conducted with interested clinics. To participate, clinics needed to have at least 15 patients with type 1 diabetes and at least 15 patients with type 2 diabetes on insulin. Clinics were invited to participate by signing a Spoke Collaboration Agreement, submitting clinic-level outcomes data, and asking PCPs to attend web-based education sessions and submit cases for case presentations. Notably, clinics were provided a stipend for participation in ECHO Diabetes.

ECHO Diabetes uses a Hub and Spoke model digitally connecting specialists (the “Hub”) with PCPs (the “Spokes”). During weekly, 1-hour ECHO Diabetes sessions conducted via secure video communication (Zoom Video Communications Inc), the Hub team presents a brief didactic topic, and the remainder of the session is for 1 Spoke to discuss a specific diabetes case from their clinic. A sample of the Project ECHO Diabetes curriculum is provided in Textbox 1. In this all teach-all learning model, PCPs from the Spoke sites were encouraged to lead the discussion by asking clarifying questions and providing their own recommendations. Only after the Spoke site PCPs met or exceeded their level of comfort in managing the case did the Hub team experts weigh in. This model encouraged independent thinking and best practice sharing among peers while still providing expert-level guidance. In addition to the ECHO Diabetes sessions, the Hub team offered real-time support to the PCPs at the Spoke sites through individual telemedicine or telephone consultations.

Perhaps most importantly and as a unique aspect of the ECHO Diabetes programs, a Diabetes Support Coach (DSC) was hired to work directly with each Spoke, its PCPs, and people with diabetes in the community. Providers were invited to identify well-managed patients from their clinic who either lived with diabetes (type 1 or type 2 on insulin) or cared for a loved one with diabetes to serve as DSC. These individuals were already familiar with the clinics and providers, and experts in their local community and its resources. Additionally, the personal connection each DSC had to diabetes was invaluable for the role. Individuals were invited to apply for these paid positions and interviewed by the Hub teams. Stanford and the University of Florida employed the DSC teams and provided supervision and specialized peer coach training by the ECHO Diabetes Hub team. Required training for DSCs included Association of Diabetes Care and Education Specialists’ Diabetes Paraprofessional Level I certification, the University of California San Francisco Center for Excellence in Primary Care’s health coach training, and all training required by the respective institutions, such as Health Insurance Portability and Accountability Act and institutional review board (IRB) trainings. DSCs met weekly with Hub teams, attended weekly ECHO Diabetes clinic sessions, and attended ongoing trainings from health psychologists and social workers. Providers could refer any patient with type 1 or type 2 diabetes (using insulin) to use a DSC though participation from the people with diabetes was voluntary. Many DSCs held hours inside of the physical clinic location, but providers could also connect people with diabetes via phone or email to DSCs. DSCs provided one-on-one support for people with diabetes seen for care at the Spoke sites, hosted regular social events (web-based and in-person), and created local resource guides specific to the community. DSCs encouraged people with diabetes as they worked toward their individual diabetes goals, and worked to address disparities in diabetes care. This unique role helped to fill a gap in health care delivery and connect people with diabetes with a trusted cultural insider from their own community to support them in their diabetes management. Challenges and limitations of the DSC model include (1) funding, as the DSCs were hired and paid by the academic institutions; (2) integration of the DSCs into external Spoke sites, as DSCs often needed extra training or certifications to be able to access electronic health records and clinic space; and (3) work-related stress and burnout as a result of this challenging role [10].

Herein, we provide a case series of people with diabetes who received Project ECHO Diabetes services, including access to DSCs, in an effort to highlight descriptive outcomes from the ECHO Diabetes intervention.

**Project Extension for Community Healthcare Outcomes Diabetes curriculum (T1D: type 1 diabetes; T2D: type 2 diabetes).**

- The Pillars of Success: Knowledge, Community, & Resilience
- Continuous Glucose Monitoring
- Glycemic Targets and Glucose Monitoring
- T2D Management in Established Atherosclerotic Cardiovascular Disease
- T2D Management in Heart Failure and Chronic Kidney Disease
- T2D Management: Promoting Weight Loss
- T2D Management: Strategies to Minimize Hypoglycemia
- Using and Interpreting Data from CGMs
- Initiating Insulin and Dose Calculations for T1D and T2D
- T2D Management: When Medication Cost Becomes a Barrier
- Types of Analog Insulin
- Initiating Insulin and Dose Calculations in T1D and T2D: A Case Based Approach
- Initiating Insulin Pump Therapy
- Introducing Diabetes Technology to Patients
- Screening for Depression and Diabetes Burnout
- Carbohydrate Counting and Dietary Management in T1D
- Making a Diagnosis of Diabetes in Primary Care
- Diabetes and Hypertension Management
- Dyslipidemia and Diabetes
- Exercise Strategies in Diabetes
- Motivational Interviewing
- Diabulimia and Disordered Eating
- Diabetes Complications and Screenings
- Sick Day Management and Severe Hyperglycemia

## Methods

### Study Design

Spokes were asked to provide short descriptions of people with diabetes who benefited either directly or indirectly from PCP participation in Project ECHO Diabetes. Cases were collected, and reports were standardized, summarized, and analyzed.

### Ethics Approval

The ECHO Diabetes intervention and assessment was approved by the Stanford University IRB (54198) and University of Florida IRB (UF IRB201800382) and conducted in compliance with the standard of Good Clinical Practice and Declaration of Helsinki. Participating Spokes and PCPs signed a “Spoke Articulation Agreement” that outlined expectations for participation, including the expectation that all cases shared are deidentified to protect confidentiality. Each Hub’s IRB granted a Health Insurance Portability and Accountability Act waiver or waiver of consent so that participating PCPs could share eligible cases as part of the project. No protected health information was included in the write-ups. Providers were given instructions to anonymize the cases and to not include any identifying information on their submissions. Research coordinators further reviewed the case submissions to ensure confidentiality was protected. Because this study was not a randomized controlled trial and was a prospective observation of patients with the intervention delivered to providers, the trial is not registered in a public trials registry.

## Results

### Overview

A total of 10 PCPs (including physicians, nurses, nurse practitioners, social workers, and Certified Diabetes Care and Education Specialists) and 1 DSC from California and Florida provided write-ups for 11 different diabetes cases between 2021 and 2022 are summarized in Table 1.

**Table 1:** Summary of cases and primary care provider interaction with Project ECHO^a^ Diabetes.

Case	Age (years), sex	Diabetes type × duration	Comorbidities	Interaction with Project ECHO Diabetes	Outcome
1	32, female	T2D^b^ × 18 years	Anxiety, depression, retinopathy, peripheral neuropathy	Introduced to CGM^c^ and educated on how to use it	HbA_1c_^d^ 11.5% to 5.3% in under 1-year, healthy pregnancy
2	63, male	T2D—new diagnosis	Hypertension, dyslipidemia, cardiovascular disease, right ischemic stroke with left-sided hemiparesis	Educated on lifestyle modifications, PCP^e^ received guidance on insulin titration	HbA_1c_ now 5.4%
3	33, male	T1D^f^ × 20 years	Polyneuropathy with a history of toe amputation, retinopathy, poor mental health, and chronic kidney disease on hemodialysis	Increased engagement, education, and fostered collaborative team approach	HbA_1c_ 14.0% to 7.8% after a year, the person with diabetes reports improved mental health and confidence
4	37, female	T1D × 26 years	Gastroparesis, chronic kidney disease, severe hypoglycemia	Helped solidify a plan to give rapid-acting insulin post meal	HbA_1c_ 10.4% to 7.6%, reports increased sense of safety
5	22, male	T1D × 8 years	Depression, anxiety, fear of hypoglycemia secondary to traumatic car accident and device mistrust	PCP education on diabetes technology resulted in person with diabetes using AID^g^	HbA_1c_ 12% to the mid-8% range in 2 years, now making insulin adjustments independently
6	48, male	T1D × 20 years	Frequent hypoglycemia resulting in safety concerns, and mood imbalance	Started on CGM, educated on how to use his CGM to help prevent lows before they happened	HbA_1c_ 7.3% to 6.4% with decreased hypoglycemia, reports improved health and QOL^h^ for himself and his family
7	63, female	T2D × 18 years	Concern for burden of insulin use, limited nutrition knowledge	Matched with a DSC^i^ who invited her to Diabetes Hour gatherings where she gained comfort with insulin and received education on diet	HbA_1c_ 8.7% to 6.7% within 2 years, feeling comfortable with insulin use
8	26, female	T1D × 14 years	Depression, anxiety, above target HbA_1c_ early in pregnancy	Matched with a DSC who helped with lifestyle modifications and improved her mental health	HbA_1c_ 12.8% to 7.1% over the course of the pregnancy, delivered a healthy baby
9	68, male	T2D—new diagnosis	Hypertension	Matched with a DSC who educated him about diet, alcohol intake, insulin use, and blood sugar monitoring	HbA_1c_ >14% to 10.8% in 1 month, now prioritizing health
10	70, male	Prediabetes × 7 years; T2D—new diagnosis	Class III obesity	Matched with a DSC and nursing case management team who educated him on physical activity requirements and medication use	HbA_1c_ 7.2% to 5.3% in 1 year, with reduction in medication doses
11	54, male	T2D × 10 years	Anxiety, hyperlipidemia, alcohol abuse complicated by acute pancreatitis	Matched with a DSC for carbohydrate counting education and general support, and was started on CGM	HbA_1c_ 10% to 7.9% within 2 years with increased health care engagement

^a^ECHO: Extension for Community Healthcare Outcomes.

^b^T2D: type 2 diabetes.

^c^CGM: continuous glucose monitor.

^d^HbA_1c_: hemoglobin A1c.

^e^PCP: primary care provider.

^f^T1D: type 1 diabetes.

^g^AID: automated insulin delivery.

^h^QOL: quality of life.

^i^DSC: Diabetes Support Coach.

### Case 1

Case 1 highlights a 32-year-old woman living with type 2 diabetes since the age of 14 years. Both of her parents died due to complications from diabetes while in their 50s. She struggled to take care of her family growing up without working parents, shifting attention away from her own health. With no health insurance, she rarely had proper health care treatment, which contributed to her worsening anger, depression, and anxiety. Prior to recent intervention, she lost vision in 1 eye due to diabetes-related retinopathy and had constant pain in both lower legs due to peripheral neuropathy. In 2021, her PCP was introduced to ECHO Diabetes, which was able to provide her with samples of continuous glucose monitor (CGM). ECHO Diabetes didactics taught the PCP how to take full advantage of CGM technology, and the PCP was able to share insights with the person with diabetes. She was also connected to a DSC through the program, who supported her with lifestyle changes and use of CGM. From April 2021 to August 2021, her hemoglobin A_1c_ (HbA_1c_) improved from 11.5% to 7.0%, without significantly increased time below range, which was the lowest HbA_1c_ she had ever achieved. She felt that the combination of CGM and peer coaching provided through ECHO Diabetes helped her optimize her glycemic management. Positive lifestyle changes continued, followed by a healthy pregnancy in late 2021. As of January 2022, she had an HbA_1c_ of 5.3% and a healthy pregnancy at 20 weeks gestation.

### Case 2

Case 2 highlights a 63-year-old man recently hospitalized for 5 days due to a right ischemic stroke. He was not previously diagnosed with diabetes and had not seen a health care provider for over 20 years. He was diagnosed with type 2 diabetes, hypertension, dyslipidemia, left-sided hemiparesis, and cardiovascular disease. The person with diabetes was also placed on 30 units of insulin (ie, 70/30 premixed insulin) twice daily. His HbA_1c_ was unknown at insulin initiation, but his blood glucose was monitored at the skilled nursing facility in which he was placed. He was uninsured and could not access endocrinology care, and relied on his PCP for diabetes management and oversight. After participating in ECHO Diabetes, his PCP felt much more confident managing diabetes. The PCP learned about tapering insulin and received feedback from the learning network on how to safely and effectively taper insulin use based on the needs of this person with diabetes. The PCP, along with the Diabetes Nurse Educator at the clinic, provided additional ECHO Diabetes supported education to help him feel empowered in diabetes management after the stroke. The PCP learned about the psychological impacts of diabetes and focused on providing patient-centered care and psychological support. The person with diabetes made several lifestyle changes to his diet, frequency of physical activity, physical therapy, and began regularly taking his medication as prescribed as a result. His insulin was titrated down on multiple occasions, and his dosage was lowered to 15 units of insulin 70/30 twice daily, after accessing real-time support from the ECHO Diabetes Hub team. His most recent HbA_1c_ was 5.4% on this regimen after 9 months of seeing this PCP.

### Case 3

Case 3 highlights a 33-year-old man with type 1 diabetes. The person with diabetes was diagnosed when he was 13 years old. He had numerous vascular complications including polyneuropathy, toe amputation, retinopathy, and was on hemodialysis. He presented with a multitude of psychosocial barriers to health care including lack of health insurance, financial hardship, food insecurity, and limited support system. He had a history of alcohol dependence, severe visual impairment, chronic pain, and poor mental health. ECHO Diabetes was especially helpful to his PCP as access to local endocrinology specialists was extremely limited in their area, and the person with diabetes had many other social barriers to accessing specialty care. The PCP used strategies learned through ECHO Diabetes including patient-centered care, motivational interviewing, a collaborative team approach, participation in case presentations, learnings from educational lectures, and real-time support with the Hub team to address medication dose adjustments and complication management. The PCP appreciated the information on psychosocial impacts of diabetes, and considered this when caring for the person with diabetes. He also began using CGM, which provided valuable data to assist with insulin dose adjustments and overall diabetes education. ECHO Diabetes gave the PCP additional skills and allowed for more active engagement and likely contributed to a marked improvement in his diabetes management skills. Prior to the ECHO Diabetes intervention, he was not engaged in the management of his diabetes and had an HbA_1c_ of 14.0%. One year following ECHO Diabetes intervention, his HbA_1c_ was 7.8%.

### Case 4

Case 4 highlights a 37-year-old woman with type 1 diabetes diagnosed at age 11 years and complicated by gastroparesis and chronic kidney disease. Her case was presented during an ECHO Diabetes clinic session when her HbA_1c_ was measured at 10.4% in June 2020. She had frequent episodes of severe hypoglycemia (<50 mg/dL), one of which required administration of glucagon by paramedics. These hypoglycemic episodes commonly occurred when she gave herself rapid-acting insulin before meals but was unable to finish the meal due to her gastroparesis. The ECHO Diabetes team helped to solidify a plan to give her rapid-acting insulin after she completed meals, which prevented over-bolusing and significantly reduced the rates of severe hypoglycemia after meals. The person with diabetes previously lacked confidence with counting carbohydrates, and based on feedback from the case presentation, the PCP also learned to empower her to bolus with a set insulin dose based on the size of the meal (eg, small meal and large meal). The discussion during the case presentation was particularly helpful to the PCP, who worked toward a creative, patient-centered solution that addressed issues related to gastroparesis specifically and positively impacted the person with diabetes. She was also connected with a DSC through the ECHO Diabetes program for peer support outside of the clinic. The DSC worked to support her around the changes implemented in the diabetes management plan, identify and support goals, and empower her around mealtime insulin boluses. She reported feeling supported by the DSC and more confident in her diabetes management. Due to the PCP’s participation in ECHO Diabetes and the connection to the DSC, the person with diabetes was able to reduce her HbA_1c_ to 7.6% by May 2022.

### Case 5

Case 5 highlights a 22-year-old man who was diagnosed with type 1 diabetes at the age of 14 years. He presented to his current PCP at age 20 years. At the time, the person with diabetes was struggling with significant depression and anxiety and had an HbA_1c_ of 12.0%. Prior intervention had failed because he had a severe distrust of diabetes devices and had a fear of hypoglycemia, exacerbated by a traumatic car accident, challenges in managing his glucose levels, and difficulty obtaining his insulin and medical support for his diabetes. After trialing an insulin pump and CGM, he was willing to discuss using an automated insulin delivery (AID) system. In February 2020, his PCP presented the case to ECHO Diabetes for assistance with the transition to AID technology. The ECHO Diabetes Spoke sites and Hub team gave the PCP recommendations to get started on a new AID system. In December 2020, he was using the system with continued distrust and fear, and his HbA_1c_ at the time was 10.6%. After further help from the ECHO Diabetes Hub team and at the request of the person with diabetes to host follow-ups every 2-3 weeks, he became more confident in his diabetes management. By June 2022, his HbA_1c_ was reduced to 7.8%, and he began making insulin adjustments independently. He reported higher job satisfaction and improved mental health with the help of his PCP and ECHO Diabetes.

### Case 6

Case 6 highlights a 48-year-old man living with type 1 diabetes for nearly 20 years. The person with diabetes lived in rural communities for the last 15 years and did not have access to an endocrinologist or diabetes technology. He often struggled with low blood sugar and mood lability. He and his wife almost lost their lives in a car accident due to a hypoglycemic event that he experienced while driving. After his PCP joined ECHO Diabetes, they felt more comfortable in prescribing CGM. Within the first month of CGM use, the device provided alarms for hypoglycemia prompting him to stop driving several times. The device also alerted him and his wife of nocturnal hypoglycemia. With the help of ECHO Diabetes and recommendations from the Hub team and Spoke sites, the PCP taught him how to use the glucose rate of change information to prevent hypoglycemia episodes before they occurred. The CGM data also provided valuable insight to the PCP into his insulin needs, and the PCP adjusted his insulin doses appropriately. Prior to receiving CGM, his HbA_1c_ was 7.3% with frequent episodes of severe hypoglycemia. His most recent HbA_1c_ was 6.4%, and the frequency of severe hypoglycemia (along with episodes of hyperglycemia) has decreased significantly. He recently reported improved QOL for his entire family and increased safety with the use of the CGM. The PCP also reported feeling more comfortable prescribing and managing CGM technology.

### Case 7

Case 7 highlights a 63-year-old woman living with type 2 diabetes, diagnosed when she was 45 years old. The person with diabetes was fearful of taking insulin because she frequently traveled out of the country and felt like it would be a burden having to transport insulin and supplies. She also felt confused about her diet and did not know what foods to eat, as she did not want to exacerbate her condition or blood glucose levels. Through ECHO Diabetes, the PCP matched her with a DSC that was able to alleviate her concerns and help her better manage her diabetes. The DSC invited her to the “Diabetes Hour” weekly web-based social gatherings and a few local social events. Through these gatherings, the person with diabetes learned how to travel with her insulin supplies and she learned about how to better manage her diet. After being in contact with her PCP and the DSC for a couple of months, she felt much better about her condition and she felt confident that she could manage it properly using new technologies introduced to her through ECHO Diabetes. Her HbA_1c_ improved from 8.7% to 6.7% after 18 months of ECHO Diabetes engagement by her PCP. She continued to be engaged with her DSC to optimize her treatment and management.

### Case 8

Case 8 highlights a 26-year-old woman living with type 1 diabetes since the age of 12 years. She started to meet with a DSC that she was paired with through ECHO Diabetes because she wanted to be healthier for her family. Six weeks after their introduction, the person with diabetes found out that she was pregnant and became very stressed about her glucose management. After working on lifestyle modifications with the DSC and her PCP, her HbA_1c_ decreased from 12.8% to 8.9% in just 8 weeks. She continued to meet with her PCP, her DSC, and her obstetrician throughout the course of her pregnancy. The DSC helped her create a plan to support a lower HbA_1c_, including discussion of healthy meal planning and regular exercise. The DSC remained in close contact with her throughout the pregnancy to remind her of upcoming appointments, accompany her to appointments, discuss blood glucose changes and ways to prevent severe hypoglycemia and hyperglycemia, and offer ongoing support. The DSC also lives with type 1 diabetes and experienced 2 pregnancies herself, which gave the person with diabetes unique insight and comfort into her own experience. She reported feeling relieved having the support of someone who could relate to her. She confided in the DSC about her past struggles with depression and reported that their relationship helped improve her mental health. At 38 weeks, the person with diabetes delivered a healthy 7-pound, 14-ounce baby. Her most recent HbA_1c_ was 7.1%, which her health care team attributed in part to their participation in ECHO Diabetes.

### Case 9

Case 9 highlights a 68-year-old man recently diagnosed with type 2 diabetes. During his last clinic visit prior to engaging with ECHO Diabetes, he had a fasting blood sugar of 415 mg/dL, with symptoms of polyuria and polydipsia, and he had lost 23 pounds. His HbA_1c_ was over 14% at the time of diagnosis, and his blood pressure was 156/84 mm Hg. His PCP matched him with a DSC who helped support him with the transition to using insulin. He received education about his diet, alcohol intake, how to test blood glucose, and how to inject insulin. Within a week, his glycemic control improved. Over the course of the next month, his insulin needs dropped. His DSC continued to advise him, and he made strict dietary changes, including abstinence from alcohol. The person with diabetes prioritized his diet and overall health, and he saw drastic changes in just the first month. Specifically, within a month of receiving ECHO Diabetes support, his HbA_1c_ decreased to 10.8%, and his insulin dose was further reduced. The PCP was confident that his HbA_1c_ would continue to improve thanks to the PCP’s participation in ECHO Diabetes and the close connection between the person with diabetes and the DSC.

### Case 10

Case 10 highlights a 70-year-old man with a history of prediabetes since 2015. He was assigned a DSC through the ECHO Diabetes program after he was diagnosed with type 2 diabetes in 2021. He had an HbA_1c_ of 7.2% at the time and had a BMI of 40 kg/m^2^. The person with diabetes had no social support system, limited financial resources, and low health literacy, which made it challenging for him to manage his blood sugar levels. After starting on a glucose-lowering medication (ie, metformin), he was next seen in the clinic 4 months later. His fasting blood glucose was 551 mg/dL with polydipsia and polyuria, and he was immediately started on insulin glargine. He was reconnected with support coaching and nursing case management and had weekly coaching calls to help manage his medications and treatment. He maintained marked improvements in his diabetes self-management and began a gentle exercise routine that included resistance band exercises and stretching at home for 15-20 minutes per day, 5-6 days per week. His weight decreased, his insulin doses were reduced, and his HbA_1c_ 9 months later had improved to 5.3%. The person with diabetes attributed his success and improved health to ECHO Diabetes and the help of his DSC.

### Case 11

Case 11 highlights a 54-year-old man who was diagnosed with type 2 diabetes at age 44 years. He had a history of anxiety, hyperlipidemia, and alcohol abuse complicated by acute pancreatitis in January 2019. Before his PCP’s clinic started participating in ECHO Diabetes, he used metformin and sulfonylureas and reported frequent hypoglycemia. His HbA_1c_ fluctuated between 9% and 10%. After starting ECHO Diabetes, his PCP matched him with a DSC for carbohydrate counting education and general support. He was also prescribed CGM and educated on how to use it by the DSC. His HbA_1c_ improved to 7.9%, and his glucose time in range improved significantly over the year since his PCP’s interaction with ECHO Diabetes. He continued to engage in his treatment and remained in contact with his DSC weekly.

## Discussion

These 11 cases volunteered by Spoke PCPs and a DSC reflect the promise of the Project ECHO Diabetes intervention and specific examples of how individuals with diabetes benefited. The principal impact of ECHO Diabetes is the education amplified from PCPs and DSCs to people with diabetes. In all cases, the people with diabetes reported increased engagement with their diabetes care team and health literacy. Those matched with a DSC not only saw clinically significant improvements in their HbA_1c_ but also reported mental health benefits. Additional impact included increased knowledge and confidence from PCPs treating people with diabetes. PCPs reported that the ECHO Diabetes didactics and case presentations, along with recommendations and guidance from the Hub team and other Spoke sites, provided value and helped them to solve problems creatively to improve care and help people with diabetes reach their goals.

While there are clear benefits for the individual participants presented herein, there are several limitations to making conclusions based on a case series. Since this case series was not a randomized controlled trial and did not include a control group, the efficacy of the overall Project ECHO Diabetes program cannot be assessed in this manner. That said, a rigorous formal analysis of patient-level outcomes is planned. The small sample in only 2 states in the United States may not be generalizable to the entire population. PCP’s interaction with the ECHO Diabetes Hub team is variable, and some writers submitted multiple cases. Cases were self-reported and selected on the basis of success as assessed by the author of the vignette, introducing a potential selection bias. Cases with limited or no change in outcomes were not submitted. It should be noted that since the inception of Project ECHO Diabetes, there have been no reported adverse events or negative impact from provider participation. Most commonly, challenges with implementation or management changes are related to the health insurance coverage and financial insecurity of people with diabetes in the rural and underserved communities that the program serves. While the writers universally attributed success to ECHO Diabetes, it is unknown if innovations in diabetes technology and pharmacology would have naturally found their way into the PCP’s practice. Moreover, we focus on quantitative A_1c_ improvements and more subjective QOL benefits.

Project ECHO Diabetes aimed to address the ethical tenets of medical care such as autonomy, beneficence, justice, and nonmaleficence. Importantly, the ECHO model has been evaluated in multiple settings, and the safety and efficacy of the approach have been uniformly supported [5]. Related to autonomy, in Project ECHO, the primary doctor-patient relationship (PCP to people with diabetes) is maintained, and providers retain care of their own patients. In an effort to support beneficence for all patients within a Spoke site, ECHO Diabetes creates learning loops to strengthen PCPs’ subspecialty expertise and effectively democratizes specialty knowledge. In support of the notion of justice, ECHO Diabetes increases equity and aims to improve health access, as many people with diabetes lack access to regular specialty care. Some of the participating Spokes are in locations many hours away from the nearest endocrinologist. One potential ethical concern is related to long-term access to the resources provided by Project ECHO Diabetes. Without consistent financial support from state and federal governments or payers, the program is unsustainable, and PCPs are left with limited or no access to the knowledge and resources of the program. Finally, Project ECHO Diabetes addressed privacy concerns regularly and ensured deidentification of case presentations.

Given the supply-demand mismatch of people with diabetes to endocrinologists and the success of Project ECHO Diabetes thus far, there are opportunities for expansion of this project to improve health equity and outcomes in diabetes care. PCPs care for a large number of people with diabetes, and more programs like Project ECHO Diabetes could improve patient outcomes with a broader reach. Nevertheless, funding for ECHO Diabetes programs will likely need to be provided by state and federal agencies or payers to ensure the long-term sustainability of these programs.

A formal evaluation of Project ECHO Diabetes is currently in progress. The study used a rigorous stepped-wedge design and will assess outcomes at the patient, PCP, and Spoke levels. A total of 872 people with insulin-requiring diabetes were recruited and consented to participate in ECHO Diabetes across 2 recruitment phases in the summer and winter of 2021 across the states of California (n=495) and Florida (n=377). The outcomes of the study will inform future directions including possible expansion of the program, gaps and limitations, and its impact on individual people with diabetes. Future directions for this program include efforts to develop a national ECHO Diabetes program to increase reach. “Super Hubs” could be developed to train other academic medical centers to act as regionalized Hubs with local Spoke sites across the country.

In summary, these 11 cases highlight (1) the benefit of PCP education, (2) PCP receptiveness and engagement in diabetes tele-mentoring or tele-education, (3) improved PCP knowledge and confidence in complex diabetes care, and (4) improved outcomes for people with diabetes after participation from PCPs in Project ECHO Diabetes programs at Stanford University and the University of Florida. To best serve the increasing population of people with diabetes, including those without access to specialty care, novel methods to disseminate knowledge, tools, and experience [11] to improve care for all people with diabetes are needed. Project ECHO Diabetes is one such method, and these 11 case studies provide personal examples of people with diabetes who saw improved outcomes after their PCP’s participation in the program. Future directions include expanded tele-education to reach more PCPs and, by extension, more people with diabetes to improve overall health outcomes.

## Abbreviations

AID: automated insulin delivery
CGM: continuous glucose monitor
DSC: Diabetes Support Coach
ECHO: Extension for Community Healthcare Outcomes
HbA_1c_: hemoglobin A1c
IRB: institutional review board
PCP: primary care provider
QOL: quality of life
